# Art Teachers' Attitudes Toward Online Learning: An Empirical Study Using Self Determination Theory

**DOI:** 10.3389/fpsyg.2021.627095

**Published:** 2021-04-06

**Authors:** Mo Wang, Minjuan Wang, Yulu Cui, Hai Zhang

**Affiliations:** ^1^Fine Arts Academy, Northeast Normal University, Changchun, China; ^2^Learning Design and Technology, San Diego State University, San Diego, CA, United States; ^3^School of Information Science and Technology, Northeast Normal University, Changchun, China; ^4^School of Media Science, School of Journalism, Northeast Normal University, Changchun, China

**Keywords:** motivation, well-being, engagement, art teachers, online learning, self-determination theory

## Abstract

The pandemic in 2020 made online learning the widely used modality of teaching in several countries and it has also entered the spotlight of educational research. However, online learning has always been a challenge for disciplines (engineering, biology, and art) that require hands-on practice. For art teaching or training, online learning has many advantages and disadvantages. How art teachers embrace and adapt their teaching for online delivery remains an unanswered question. This research examines 892 art teachers' attitudes toward online learning, using learning environment, need satisfaction, mental engagement, and behavior as predictors. Structural equation modeling was used to explore the relationship between these four dimensions during these teachers' participation in an online learning program. The results reveal significant correlations between the learning environment, need satisfaction, mental engagement, and behavior. Moreover, this study reveals the group characteristics of art teachers, which can actually be supported by online learning programs. These findings provide insights into how art teachers view and use online learning, and thus can shed lights on their professional development.

## Introduction

Teachers must adapt to new technologies, more so than those in many other occupations. In many countries, the reform and development of education policies require continuous professional development for teachers as a way of improving the educational quality (In de Wal et al., 2020). The caliber of teachers plays an important role in promoting the reform and reconstruction of education (Vermunt and Endedijk, 2011). Moreover, teachers are instrumental in improving student performance (Zhang et al., 2020). However, teachers are often overloaded with work, and many of them need the necessary training in using new educational technologies. An investigation found that fewer than 5% of teachers believe that they have received full training, and only 36% of teachers think that they have been adequately supported (Promethean, 2020). The *Education Policy Institute of England* reported that teachers spend an average of 4 days per year on teacher training and professional development, compared with 10.5 days spent by teachers in 36 other countries (Baker et al., 2019). These studies seem to suggest that many teachers might not be prepared to teach or learn online.

Teacher training has been conducted in different ways, from in-person, to online, to hybrid. In-person training has been the dominant format until recent years. However, it is found that in-person training does not have significant influence on teaching beliefs and teaching innovation. It also have not generated long-term and lasting impact on pedagogy (Duncan-Howell, 2020). By contrast, in online training, teachers can participate in the same course anywhere and anytime, and can conduct more meaningful discussions with their peers through collaboration and social media tools. At present, online learning has become a mainstream method of education in many countries (Kara et al., 2020) and is becoming a very important learning tool for teachers' professional development (Zhang et al., 2017). Teachers welcomed online learning for its flexibility, autonomy, opportunities for personalized learning and peer interactions (Chieu and Herbst, 2016). Additionally, online learning can promote the sharing and co-construction of the educational experience through effective social interaction (Kent et al., 2016). Hence, online learning is considered an important way to develop teachers' thinking skills and to promote their professional development (Quinn et al., 2019).

When considering the effectiveness of teacher online training, we inevitably pay much more attention to the psychological mechanism of teachers' learning process including mental engagement. It is necessary to have a deep understanding of this psychological mechanism because it can help us understand the effects of online learning and how it influences online behaviors (In de Wal et al., 2020). This can help trainers to systematically intervene and guide teachers' online learning. Among the psychological attributes that influence teachers' professional learning, the most important one is motivation, according to self-determination theory (SDT) (Gorozidis and Papaioannou, 2014). Researchers believe that self-determination helps people stay motivated and can direct and predict their performance (Jeno et al., 2017). It can also guide their learning engagement and behavior (Reeve, 2006). When learners' psychological needs are met, they show enhanced motivation and sense of well-being (Jeno et al., 2019a). These psychological needs arise from the individual's perception of the learning environment (Deci and Ryan, 2000), and the environment also influences the psychological mechanism of learners' participation in the learning process. Ultimately, mental engagement influences learning behaviors and participation in the learning process.

**Figure 1:**
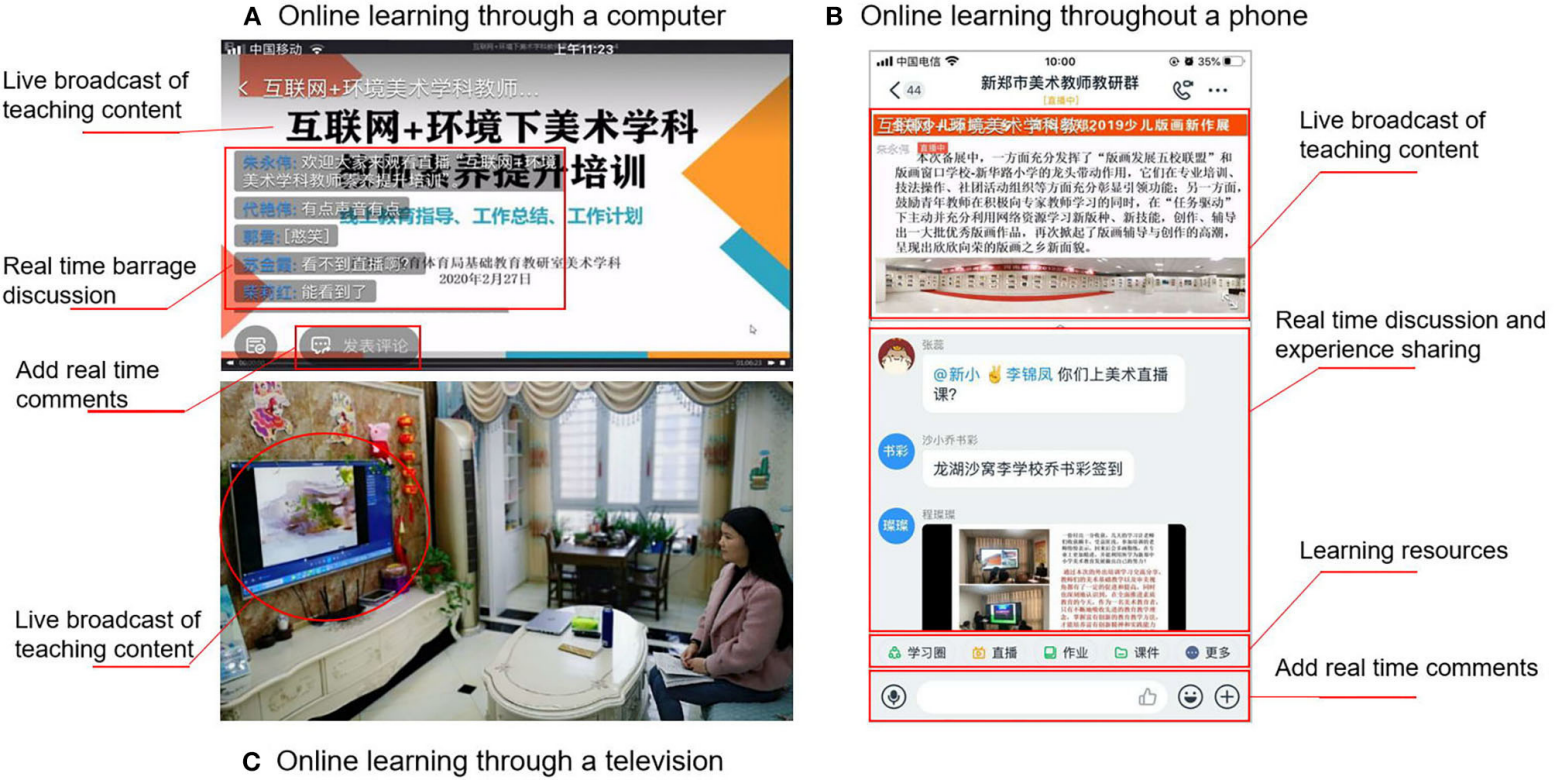
Three ways for teachers to participate in an online learning program.

In China in particular, the application of technology to the field of education not only has an impact on the major disciplines such as mathematics and English, but also on art education in K-12 schools. For example, the online learning of Mona Lisa Smile enables participants to enjoy master's work and enhance professional appreciation skills. Art teachers can use online platforms to zoom in great works and carry out detailed instructions. They can also exchange ideas through online platforms, which promotes teachers' active thinking and participation. This platform promotes interactive learning experience by creating cooperation and communication among this group of teachers (Hiltz, 1995). In February 2020, Henan Province in China, launched an online training program for art teachers, whereby they could participate in training activities via mobile phones, computers, or televisions at home (see Figure 1 for details). Through this process, teachers not only learned subject knowledge, but also enhanced their experience through live online courses and peer sharing, effectively developing their professional ability and skills (Qihan, 2020).

Overall, art education is a discipline to cultivate visual ability and innovation skills in primary and secondary schools. Online learning for art teachers is also important because of its mediation in transfer specific contents through all kinds of information devices (Yang, 2000). Online learning has become an important way for teachers' self-development in that teachers can learn flexibly and independently. During the COVID-19 pandemic, this form of online learning has become more common in China. It can promote the integration of technology into art discipline, helping art teachers go beyond the limitations of traditional art instructions (Yang, 2000). However, due to the hands-on requirement of art education, how art teachers can train through an online learning environment requires further investigation. This study fills in this void, by assessing the relationships between the online learning environment and art teachers' basic psychological needs, mental engagement, and behavior after their participation in an online training program. This study can also help teachers better understand factors influencing their online learning performance, so as to improve their own learning outcomes.

### Self-Determination Theory

Self-determination theory (SDT) is a macro-level theory about human motivation and wellness that contains six mini-theories to explain the relationship between motivation and basic psychological needs (Deci and Ryan, 1985). In SDT, Basic Psychological Needs Theory is one of them and contributes to motivation and well-being (Ryan and Deci, 2017). Researchers believe that the satisfaction of three general psychological needs of SDT, namely, autonomy, competence, and relatedness plays important roles in intrinsic motivation and better well-being (Ryan and Deci, 2000). According to Garn's elaboration, autonomy refers to the basic need for self-organization and regulation of behavior in line with one's self-consciousness; competence means the need to develop personal abilities and skills and to interact effectively with the environment; relatedness is the need to feel socially connected in a learning environment (Garn et al., 2019). Nowadays, SDT is applied in various fields, such as the workplace, commercial services, educational services, and is considered by researchers to be one of the “most empirically supported motivation theories” (Sun et al., 2019).

In educational research, SDT also has a wide range of applications. In studies of massive open online course (MOOC), SDT can also contribute by exploring how the three basic psychological needs (i.e., autonomy, competence, and relatedness) influence intrinsic motivation and final behavior in the process of online learning (Sun et al., 2019). Researchers also used SDT to explore the influence of mobile learning tools on learners' motivation, achievement, and well-being (Jeno et al., 2019a). In online learning environment, teachers are also learners of various learning resources. When their basic psychological needs are met, learning behavioral intentions toward learning also become more pronounced from the perspective of SDT (In de Wal et al., 2020). This indicated that SDT explains the psychological attributes and mental engagement of the process of teachers' professional learning and is essential for teacher development research. But it is still a complex system for teachers' professional learning and the change of environment may influence teachers' psychological process. For example, in a study of virtual learning environments, researchers discovered that 3D virtual reality technology contexts can meet or hinder specific psychological needs of learners, which in turn influences learners' participation and behavior (Huang et al., 2018). Various technologies can also boost teachers' professional development.

### Online Learning Environment and Technology

Emerging technologies in recent years, such as tablets, smart phones, laptops, and other electronic smart devices, have initiated a new trend in the field of education and have enriched teaching and learning (Huda et al., 2018). On this basis, online learning has gradually entered educational practice and research. Online learning environment is an open and distributed learning environment supported by the Internet and other technologies, such as telephone, videotape, satellite transmission or computer (Zuhairi, 2018), using these techniques to mediate a necessary communication (Jonassen, 2004). This kind of learning can be both in synchronous or asynchronous format. The former requires that learning take place at a fixed time, similar to traditional offline learning in a fixed place; the latter is relatively flexible on timing, allowing self-paced learning and the exchange of resources over a communication network (Khan, 1998). No matter being in what format, online learning has advantages over face-to-face learning, such as balancing educational resources and development, reducing costs, and promoting educational equity (Johnson and Aragon, 2003).

Bates believes that the main reason why online learning is applied widely is not only to improve education and training opportunities and learning quality, but also to reduce costs and to improve effectiveness (Bates, 1997). Compared to traditional offline learning, online learning is full of many factors influencing online learning engagement (Montgomerie et al., 2016), which requires learners to be more self-disciplined. Learning online may also change teachers' assumptions and beliefs, as well their practice of face-to-face teaching (Huppert, 2009). Another important influence is that online training programs can change teachers' understanding of teaching. In other words, even a short online training course may influence teachers' understanding of various situations, especially for new teachers who may have a lack of teaching experience and skills (Vilppu et al., 2019). This not only places greater responsibility on online learners, but also creates more opportunities and possibilities for teaching practice and innovation.

Online learning has proved its effectiveness in teacher training programs, especially for pre-service teachers (Archambault et al., 2014; Luo et al., 2017), however, it still faces many challenges. Online learning is not an ideal learning environment that can deliver a perfect learning context outside of the classroom, as is described by *American English* (https://americanenglish.state.gov/resources/teachers-corner-online-learning). Norton and Hathaway (2008) proposed that pedagogical models need to be further explored and designed in online learning environment. The ability and professionalism of online learning teachers also gained more concerns from the leadership when facing on state-licensure tests or assessments (Hurlbut, 2018). Although there are still challenges in online learning, it is regarded as an effective way of distance learning. Especially during this pandemic, teachers had to accept online learning as the only training measure and might become more resilient in future similar situations (Zhu and Liu, 2020).

### Mental Engagement

Mental engagement can be seen as a kind of mental state and mechanisms in information processing (Ben Khedher et al., 2019). Well-being is part of these psychological mechanisms and is considered a combination of feelings that can support work performance. Huppert posited that well-being encompasses happiness, interest, participation, self-confidence, and other emotions that influence the development of potential abilities and control over human life (Huppert, 2009). Whether being positive or negative, the long-term experience of certain emotions leads to the formation of a particular subjective judgment, which influences our physical or mental health (Brackett and Baron, 2018). As described by the *Organization for Economic Co-operation and Development* (OECD) *Education and Skills Today*, in complex societies and economies, the relationship between teachers' well-being and teaching should also be the focus of research when the previous literature shows that teacher's well-being can impact their effectiveness of teaching (Fraser, 2020). Better evidence-based interventions are also needed to promote teacher's well-being (Taylor and Francis, 2019).

Human experience is influenced by various psychological mechanisms, where satisfaction is also an important dimension in mental engagement like well-being. Satisfaction is a concept that comes from the business field and involves customers' intentions to purchase goods or to spread a positive attitude toward goods and services. In education, understanding learner satisfaction is the best way to assess the effectiveness of the training program (Aimsrikul and Thitinaruemit, 2019). For online education, teachers are generally satisfied when they receive good institutional support (Stickney et al., 2019). For online art learning, satisfaction influences teachers' willingness to participate in training and learning, and their skills will also improve. Previous research has highlighted that the mental engagement of teachers is closely related to their working environment and motivation (Trucchia et al., 2013; Barbieri et al., 2019). Studies have shown that teachers' well-being and satisfaction are positively related to work performance, and the same is true for art teachers. This indicated mental engagement contributes to active involvement in the online learning process (Sun et al., 2019).

## Methodology

### Participants

Participants of this study are 892 art teachers (87.60% female) from different primary and high schools in Jilin Province, China, and most of the teachers (62.6%) were under 40. The 29.83% teachers graduating from Second-tier Teacher Education Institutions (a degree that is slightly lower than a bachelor's degree and is equivalent to a teaching credential). The majority (56.4%) holding a Bachelor's degree, while 13.8% had a Master's degree or higher. Over 46% of the teachers reported that they had been teaching for 10 years, while 29% of the teachers were new. Over half of the teachers (60.7%) stated that they had good experience of online learning, while 77% of the teachers reported that they had completed <3 h of online learning per week. See Supplementary Table 1 for demographic details.

In August 2020, these teachers participated in a 1-week online training program. The training program was initiated by the municipal Basic Education Research Center (BERC). The theme of the program was “Autumn Online Training Program for Teaching Materials of Arts.” The content of the training program included four parts: (1) a summary of the online teaching experience during the pandemic, (2) experience and achievement in sharing art education, (3) observation and learning of excellent teaching cases, and (4) online communication and discussion between teachers. In this program, teachers should not only have learned from excellent courses and cases through online learning platform, but also jointly initiate discussion through online communication tools, sharing their learning experience to build an online learning community of teachers.

### Measures

There were four dimensions to the questionnaire, namely, the environment, need satisfaction, mental engagement, and behavior, including eight subscales. Each subscale was adapted from a part of the questionnaires designed by Jeno et al. (2019b), Ryan et al. (2006), Valtonen et al. (2017), Watson and Clark (1999), Keller (2017), and Huang et al. (2018). These questions were revised to conform to the characteristics of online learning and Chinese educational culture. The teachers responded to questions about online learning using a five-point Likert scale ranging from 1 (strongly disagree) to 5 (strongly agree). See Supplementary Table 2 for the subscales and Supplementary Material for the complete questionnaire.

The **Environment** scale was designed to explore the participants' perceptions of a novel online environments and their abilities in such online learning contexts. It contained two subscales, namely, novelty (α = 0.87) and technology. The former subscale was developed based on technology adoption and innovation-related theories. Novelty may contribute to increased engagement, and predict higher interests (Adachi et al., 2017; Jeno et al., 2019b), and a seven-item scale was formed to measure such perceived novelty of online learning environments based on Jeno's work; The latter subscale employed a one-item subscale “technology competence” (Jeno et al., 2019b), and a four-item subscale “technological knowledge” (α = 0.88) by Valtonen et al. (2017), to evaluate the knowledge and skills of participants in an online learning environment.

The **Need satisfaction** scale employed *Player Experience of Need Satisfaction scale* (Ryan et al., 2006), including three subscales—perceived competence (α = 0.83), perceived autonomy (α = 0.89), and perceived relatedness (α = 0.84)—to measure the basic psychological needs of participants (Jeno et al., 2019b). After combining the characteristics of online learning, the original questionnaire was modified to suit a Chinese educational cultural background. Three subscales, namely, perceived competence, perceived autonomy, and perceived relatedness, are modified based on this work. An example of the included items is “I feel competent at online learning.”

The **Mental engagement** means learners' active participation in online learning activities with positive or negative emotions. In this study, two subscales, i.e., well-being and satisfaction, were employed to measure the mental engagement of online learners. Although the well-being subscale included general positive influence (α = 0.94) as designed by Watson and Clark (1999), researchers deleted some variables that were unrelated to online learning. For example, the feeling for online learning cannot be explained as “alert” and “strong” due to cultural differences. Then, teachers were asked to indicate what extent they have felt about online learning on a five-point Likert scale ranging from 1 (not at all) to 5 (extremely). The satisfaction subscale was modified by “satisfaction” (α = 0.82) from the Instructional Materials Motivation Survey (IMMS), a widely used questionnaire designed by Keller (2017).

The **Behavior** subscale originates from “behavioral intention” (α = 0.89), a four-item subscale that was developed to test the behavioral intentions of art teachers in further online learning by Huang et al. (2018). An example of the included items is “Wanted to find out more information about online learning.”

### Procedure

The questionnaire was designed through an online platform (https://www.wjx.cn). The municipal Basic Education Research Center (BERC) administered the questionnaire to ensure a high response rate. After a 1-week online training program, teachers were asked by BERC to answer each question via a phone or a computer. The researchers also gave a short introduction to inform the participants of the main purpose of this study and that the results would only be used for scientific research, and no personal information would be disclosed. Thus, teachers could safely share their true opinions and perspectives.

Guided by the random sampling method, six teachers were randomly selected from the subjects for interview to further understand the views and feelings of primary and secondary school teachers on learning environment, need satisfaction, mental engagement and behavioral intention after the project. The interview questions mainly focused on the above four dimensions, namely, environment, need satisfaction, mental engagement, and behavioral intention, with semi-structured questions to ask teachers about their views and feelings on these dimensions. One such example is “How do you feel about online learning? Is online learning easy to use?” Other questions addressed the reasons for online learning, for example, “Would you like to introduce or share this training experience with others? Why?”

The interview was mainly conducted through face-to-face, supplemented by phone and video conferencing. Firstly, six teachers were interviewed by face-to-face, and the whole process of this semi-structured interview was recorded in audio or video. After the interview, we converted the audio or video to texts and conducted systematic content analysis. We conducted more follow-up interviews with these teachers, to ensure our correct understanding and interpretation of data.

### Research Hypotheses

**Figure 2:**
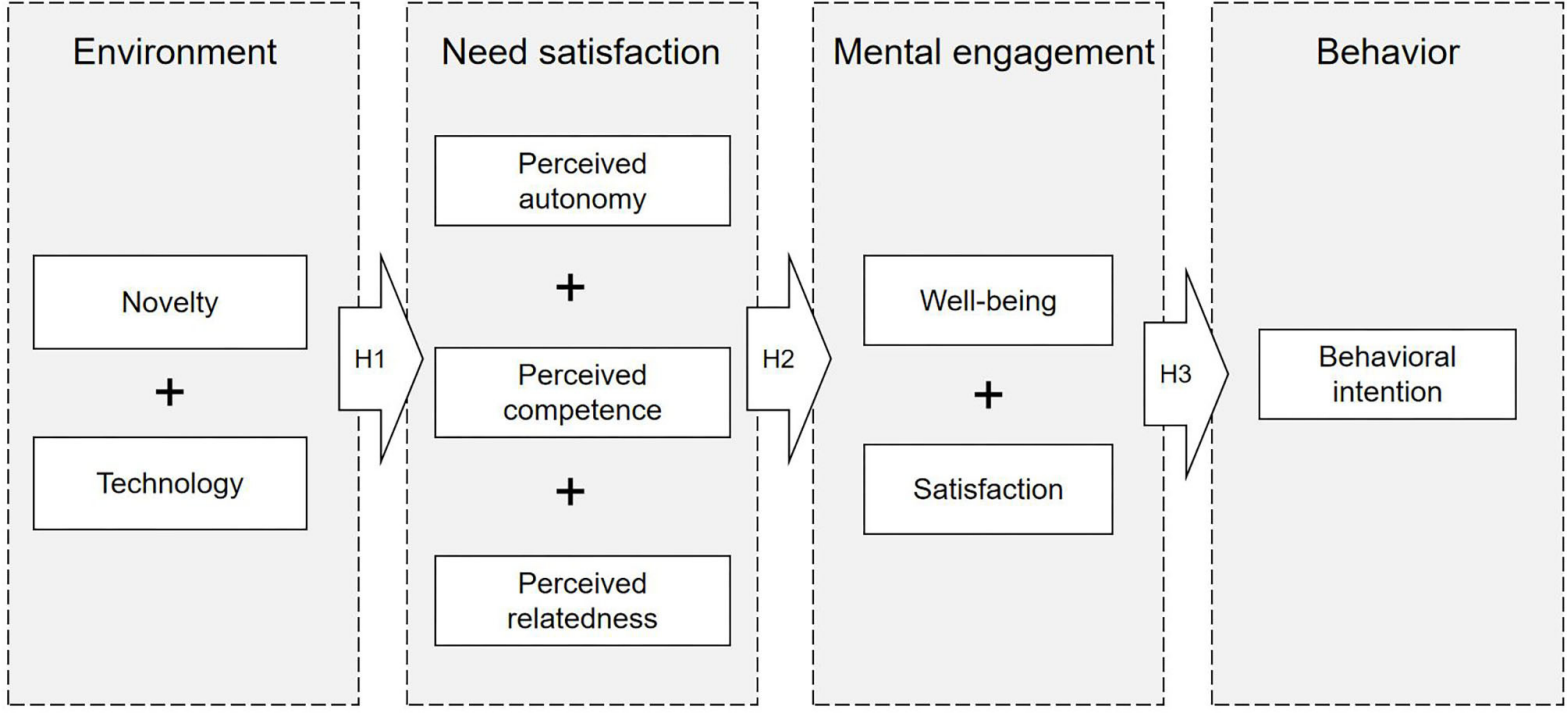
The structure of the research hypotheses.

In this study, we examine the relationships of four dimensions—learning environment, need satisfaction, mental engagement, and behaviors as outlined in self-determination theory. The overarching hypothesis is that online learning environment influences teachers' psychological state (need satisfaction and mental engagement), and then the psychological state will influence teachers' behaviors (behavioral intention). Among psychological states, basic psychological needs of the teachers are produced in a specific learning situation, and directly influences how teachers choose and use technology, media, and resources for effective online learning. Therefore, teachers' basic psychological needs may be influenced by such a learning environment (H1). Meanwhile, need satisfaction probably influences teachers' sense of mental engagement, especially their sense of well-being and satisfaction (H2). Ultimately, their behavioral intention toward online learning may also be influenced by such mental engagement (H3). Figure 2 shows the structure of these hypotheses.

## Results

### Descriptive Statistics and Reliability

**Figure 3:**
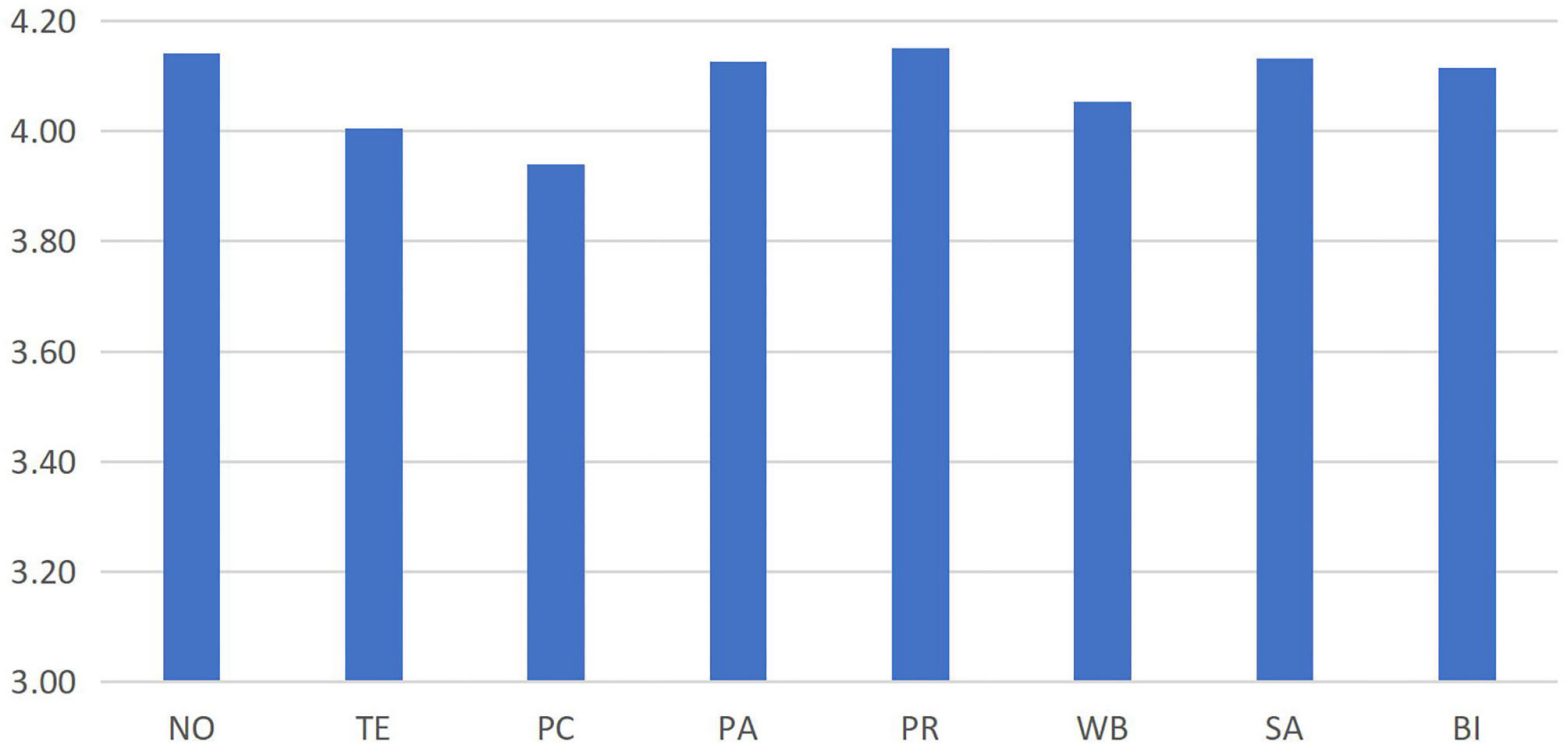
Statistical mean values of the subscales. NO, novelty; TE, technology; PC, perceived competence; PA, perceived autonomy; PR, perceived relatedness; WB, well-being; SA, satisfaction; BI, behavioral intention.

First, SPSS was used for the descriptive statistics and reliability analysis for the various subscales. As shown in Supplementary Table 3, the mean value of each subscale ranged from 3.94 to 4.15. Overall, teachers assigned relatively high scores to environment, need satisfaction, engagement, and behavioral intention (with the average > 4), which indicates that they were engaged in the online learning environment, showing greater psychological need satisfaction and higher participation. For example, in a novel environment, teachers assigned higher scores for novelty (average = 4.14), while the value of teachers' feedback in technology was relatively low (average = 3.94) as is shown in Figure 3.

The reliability shows the consistency and stability of the measurement data, as displayed in Supplementary Table 3. The results from each subscale reflect high reliability, ranging from α = 0.954 to α = 0.975—much higher than the accepted value of α = 0.8. The overall reliability is very high (α = 0.982), indicating the trustworthiness of data collected. The Cronbach's α test results of each variable are also included in Supplementary Table 3.

### Structural Model

Next, we used structural equation modeling (SEM) to investigate whether the final model was consistent with the hypotheses (Lei and Wu, 2017), with AMOS being the statistical software used for testing. We first examined fitting indices. Supplementary Table 4 shows the model fit indices that reflect the model quality. According to the standard indices (Supplementary Table 4), such as CMIN/DF, the adjusted goodness of fit index (AGFI), standardized root mean square residual (SRMR), the normed fit index (NFI), the comparative fit index (CFI), and the incremental fit index (IFI), summarized by Arpaci and Mustafa (Arpaci and Baloglu, 2016), all statistics can reach an acceptable level, indicating that the model's fitting degree is relatively high, reliable, and has high explanatory power.

**Figure 4:**
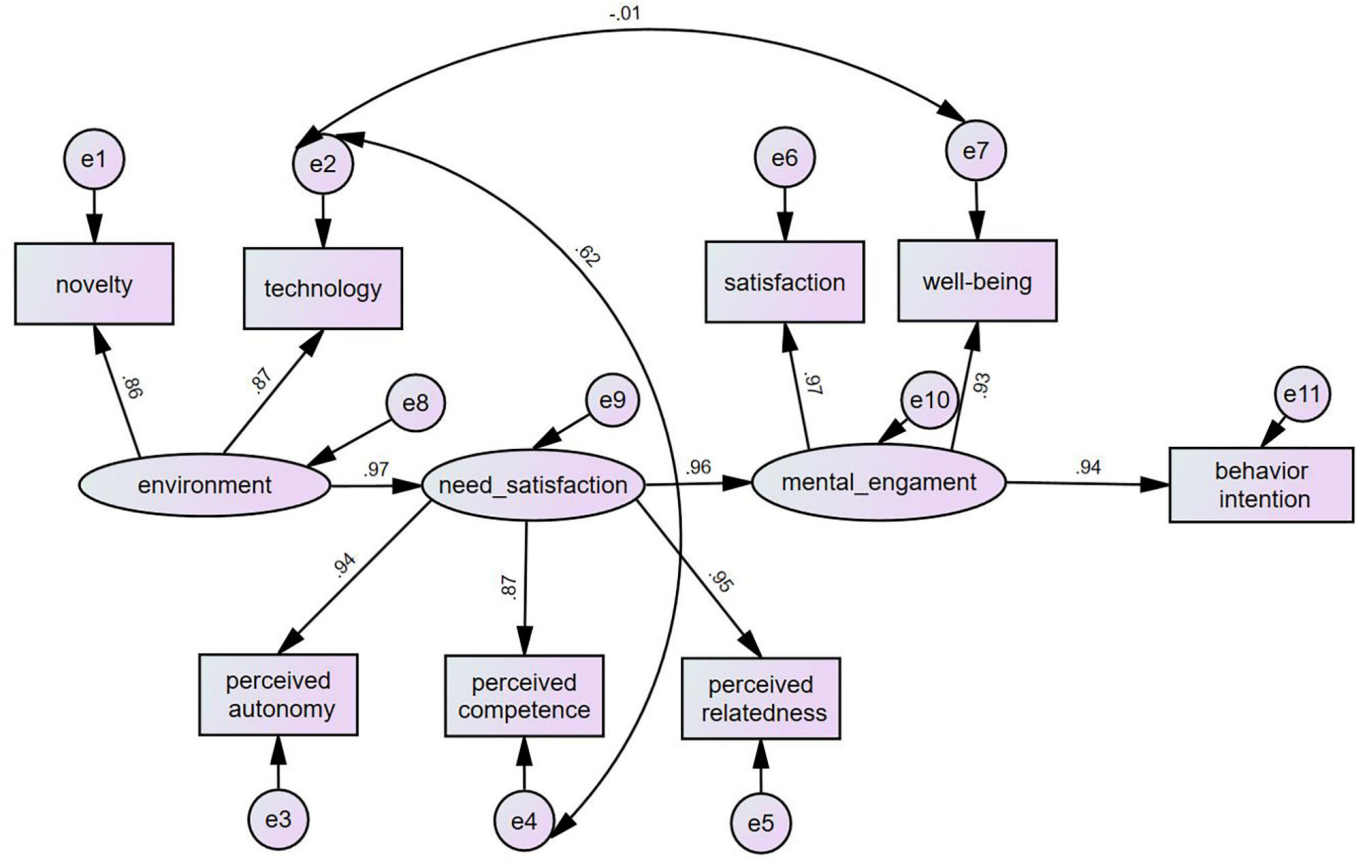
Structural model of the proposed hypotheses.

Then, path analysis proceeded with the final model. We examined structural path correlations among the environment, need satisfaction, mental engagement, and behavioral intention as specified in the hypothesis. The mean value of each subscale is calculated for comparison and is an observable variable itself. For example, in “Perceived autonomy,” there are three sub-questions and the mean value of each question is considered one observable variable. In Figure 4, there are eight observed variables, including BI, TE, PA, PC, PR, SA, WB, and NO; three latent variables, including mental engagement, need satisfaction and environment. These variables cover full aspects that required to be measured from the four aspects in research hypotheses. The overall correlation estimates are displayed on each path. These standardized estimates explain the predictive power of variables: The closer to 1.0, the greater the predictive power and the higher the correlation (Karbakhsh and Safa, 2020). The results show that learning environment as defined in this study can influence learner need satisfaction in significant ways (H1, β = 0.96, *p* < 0.01), need satisfaction of the art teachers significantly predicted mental engagement (H2, β = 0.95, *p* < 0.01), and mental engagement significantly predicted final behavioral intention (H3, β = 0.94, *p* < 0.01). These results are consistent with our hypotheses, which means that there was a significant direct effect of the environment–> need satisfaction, need satisfaction–> mental engagement, and mental engagement –> behavioral intention.

### Interview Analysis

In the interview process, most teachers provided positive responses to all questions, which piqued researchers' curiosity about the underlying reasons. After a careful inquiry into their feelings and views, we made several interesting discoveries by content analysis of the data. First of all, the online learning environment gained their attention and promoted teachers' professional development, as described by teacher T1: “I seldom had the opportunity to study online before. This training program has opened up a lot of research methods for us, which is quite interesting for me.” Similarly, teacher T3 believed that learning online is helpful because “It contains a lot of useful knowledge, and specific contents can be fed back to our own daily teaching”, which both T1 and T2 agree with. Clearly online training has enhanced these teachers' internal motivation. Secondly, teachers showed little concern about their competency with online learning. Teacher T6 said, “I often encounter problems during online learning—for example, the sound stops working. Maybe I need a helper to solve it.” Even if there were some problems, teachers were still confident about online learning, with statements like “I feel competent when I exchange my ideas with my colleagues” (teacher T3) and “I can find much useful information that I can trust, and that inspired my teaching”(teacher T6). Furthermore, art teachers reported a high level of well-being and satisfaction with online learning programs; for example, “I really enjoyed studying here with my friends, especially in this special period, and it's a good way to promote my vision of teaching” (teacher T5).

## Discussion

This research used self-determination theory (SDT) to explore the relationships between the learning environment, need satisfaction, mental engagement, and behaviors for art teachers in online learning. The results of the study revealed several interesting findings.

First of all, art teachers surveyed in this study showed several unique group characteristics. Data indicated that a great majority of the art teachers are under 40 years old, which might be due to the need for reducing communication gaps with their students. Since the research area was limited to Jilin Province, China, these findings may have been influenced by the regional cultural background. The second feature is the imbalance in the gender distribution of art teachers. Female teachers account for 87.60%, and fewer male teachers participate in online learning. The importance of gender concerns is that gender congruence between teachers and students has some effect on students' achievement, which has been attracting research attention in recent years (Doornkamp et al., 2019). In general, male learners may be more motivated by challenges and higher self-efficacy comparing Bachelor's degree with female learners (Law et al., 2010). Additionally, most of the teachers had a Bachelor's degree (56.40%) or below Bachelor's degree (29.8%), and only 13.8% teachers had the Master's degree or above. It is reasonable to be concerned with their educational background, because learners with low levels of education may encounter more course content obstacles, and higher educational level learners are less likely to experience these barriers (Henderikx et al., 2019). Thus, professional development is much needed for the majority of the art teachers.

Second, the environment, need satisfaction, and mental engagement of online learning are important factors that influence teachers' behavioral intention, and this hypothesis was verified through structural equation modeling with AMOS. Findings are presented in the following orders: (1) Teachers' need satisfaction is directly influenced by the learning environment. Need satisfaction is a complex concept that can be determined intrinsically and externally (Ryan and Deci, 2000; Peechapol et al., 2018). This means both intrinsic and external environment can regulate learners' internal learning state. In the process of online learning, art teacher T4 responded that online education “has unique innovation and technical attributes, and teachers can experience the process of art creation through technology-enhanced visualization.” The change of environment inspired teachers with more willingness and greater motivation to participate in online learning programs. (2) Teachers' need satisfaction for learning significantly influences their mental engagement, including their satisfaction and well-being (positive aspects). Milyavskaya and Koestner (2011) found that learner satisfaction is strongly related to autonomous motivation and well-being in many domains. The study further verified the relationship among the basic psychological needs. The results show that teachers can arrange the learning process in their own way (autonomy), can feel competent at online teaching (competence), and can generate positive connections (relatedness). The satisfaction of these three basic psychological needs at the same time increases their motivation toward learning and their sense of well-being (Poulou, 2020), resulting in their high level of satisfaction with online learning. (3) The satisfaction of basic psychological needs is an important factor influencing teachers' mental engagement (Karbakhsh and Safa, 2020). Teachers' mental engagement significantly influences their behavioral intention, especially when the teacher has a sense of well-being and satisfaction. Therefore, the most direct way to encourage teachers' active learning is to enhance their well-being and satisfaction and to stimulate their positive internal psychological mechanisms through an effective online learning environment.

Third, this study reveals that an online learning program is an important way for art teachers to develop their content knowledge. The community of online learning is formed among learners, and the interaction among these learners is significantly increased, which leads to higher self-efficacy and satisfaction (Cho and Cho, 2017). In the online learning program studied here, teachers can not only participate in online learning activities but can also actively reflect on their learning and form a community to exchange experiences. For example, teacher T6 considered online learning to consist of “essential learning tools that can bring out innovative ideas about art teaching.” The online learning environment is also considered a relatively new method for them that can effectively improve their teaching and learning experience. Moreover, the art teachers' responses to most questions were relatively positive, such as “really interesting,” “helpful for me,” and “want to recommend,” as stated by many teachers. In such a learning environment, teachers can also express themselves and receive recognition of successful from each other (Zhang et al., 2012). Thus, art teachers in this study can embrace online learning and perceive it as an effective boost for their professional growth.

## Conclusions

When assessing the effectiveness of online learning, we must consider how teachers experience the learning environment and the changes it makes to their psychological mechanisms, such as the correlations between the environment, need satisfaction, mental engagement, and behavior. In this paper, the structural equation modeling method was used to verify the interactions between the four dimensions and discovered significant correlations. On one hand, these findings provide a good explanation for how art teachers participate in online learning, and also reveal the attitudes of these teachers in an online learning environment from the perspective of the self-determination theory. On the other hand, the research verifies the effectiveness of online learning for art teachers, and shows how teachers perceive this novel learning method. Our research findings might help others understand how art teachers participate in an online learning environment, and to improve training programs for online teachers. These results show that optimizing online learning environments, supporting teachers' basic psychological needs and mental engagement can significantly enhance their professional development.

The study is conducted when COVID was spreading around the world, which may have influenced the participants' mental state and their attitudes. In addition, the study used random sampling method and selected 6 teachers for interviews. Even though the survey sample size is large (892), the interview sample size is also relatively small due to the pandemic. We plan to conduct follow-up studies in the near future, with a larger sample and also to track teachers' long-term changes after their participation in online training. In the following study, more samples are expected to enhance the accuracy and universality of this study. Tracking research is also needed to find out the changes after long-term teacher training programs for art teachers.

## Data Availability Statement

The original contributions presented in the study are included in the article/Supplementary Material, further inquiries can be directed to the corresponding authors.

## Ethics Statement

The studies involving human participants were reviewed and approved by the municipal Basic Education Research Center of Changchun. Written informed consent to participate in this study was provided by the participants' legal guardian/next of kin.

## Author Contributions

MoW carried out formal analysis, investigation, methodology, and writing original draft preparation. MiW thoroughly edited the original manuscript to ensure that it reaches the standard of publishing. YC carried out writing-original draft preparation, visualization, and and software. HZ carried out project administration, supervision, and resources. All authors contributed to the article and approved the submitted version.

## Conflict of Interest

The authors declare that the research was conducted in the absence of any commercial or financial relationships that could be construed as a potential conflict of interest.
