# Supplementary material for: Art Teachers' Attitudes Toward Online Learning: An Empirical Study Using Self Determination Theory
Source: Front Psychol. 2021 Apr 6;12:627095. doi: 10.3389/fpsyg.2021.627095 (PMC8083004; doi:10.3389/fpsyg.2021.627095)
Supplement: Supplementary file 1 [file Table_1.pdf]

Supplementary Material

1 Tables

Table 1. Demographics of the participants.

Variables	Category	Frequency	Percent	Variables	Category	Frequency	Percent
Age	20–29	293	32.80%	Gender	Male	111	12.40%
	30–39	266	29.80%		Female	781	87.60%
	40–49	281	31.50%	Online experience (1 = little, 5 = a lot)	1	38	4.30%
	>50	52	5.80%		2	95	10.70%
Educational background	Below Bachelor's degree	266	29.80%		3	375	42%
					4	212	23.80%
	Bachelor's degree	503	56.40%		5	172	19.30%
	Master's degree	120	13.50%	Online learning time (per week)	<1 h	107	12%
	Above Master's degree	3	0.30%		1–2 h	313	35.10%
Teaching experience	1–3 years	259	29%		2–3 h	259	29%
	3–10 years	221	24.80%		3–4 h	108	12.10%
	>10 years	412	46.20%		>5 h	105	11.80%

Table 2. The subscales of the questionnaire.

Dimension	Subscale		Cronbach's α
Environment	Novelty		0.84
	Technology	Technological competence	N/A
		Technological knowledge	0.88
Need satisfaction	Perceived competence		0.83
	Perceived autonomy		0.89
	Perceived relatedness		0.84
Mental engagement	Well-being		0.94
	Satisfaction		0.82
Behavior	Behavioral intention		0.89

Table 3. The descriptive statistics and reliability of the subscales.

	Minimum	Maximum	Mean	Std. deviation	Variance	Cronbach's α	*N* of items

	Statistic	Statistic	Statistic	Std. error	Statistic	Statistic		
Novelty	1	5	4.14	0.02	0.72	0.53	0.943	7
Technology	1	5	4.01	0.03	0.75	0.56	0.949	5
Perceived competence	1	5	3.94	0.03	0.77	0.59	0.953	5
Perceived autonomy	1	5	4.13	0.02	0.70	0.49	0.946	3
Perceived relatedness	1	5	4.15	0.02	0.68	0.47	0.962	3
Well-being	1	5	4.05	0.02	0.73	0.54	0.974	7
Satisfaction	1	5	4.13	0.02	0.71	0.50	0.975	6
Behavioral intention	1	5	4.12	0.02	0.71	0.50	0.954	4
Total							0.982	40

Table 4. Model fit indices.

	Acceptable Value Standard	Model	Results
CMIN		34.156	
CMIN/DF	<3	2.440	Accept
Goodness of fit index (GFI)	>0.90	0.991	Accept
Adjusted goodness of fit index (AGFI)	>0.80	0.977	Accept
Standardized root mean square residual (SRMR)	<0.05	0.0062	Accept
Normed fit index (NFI)	>0.90	0.997	Accept
Comparative fit index (CFI)	>0.90	0.998	Accept
Incremental fit index (IFI)	>0.90	0.998	Accept

2 Supplementary Questionnaire

Basic Information:
Age: 20–29, 30–39, 40–49, >50
Gender: Male, female
Educational background: Below Bachelor's, Bachelor's, Master's, above Master's
Teaching experience: 1–3 years, 3–10 years, >10 years
Online learning experience: 1, 2, 3, 4, 5 (1 = little, 5 = a lot)
Online learning time: <1 h, 1–2 h, 2–3 h, 3–4 h, >5 h

Questions:

Dimension	Questions
Novelty	I think online learning is a new experience for me.
	I think online learning is a new way to improve my major.
	I think online learning is an effective way to improve teaching.
	I think the online learning platform is easy to use.
	I think online learning is very interactive.
	I think it is fun to take an online learning program.

	I think the online learning courses are diversified and helpful in content.
Technology	I am competent in the technology used in online learning.
	I can solve technical problems encountered in online learning.
	I am familiar with the new technology and characteristics of online learning.
	I can use this new way to learn knowledge.
	I know several websites about online learning.
Perceived competence	I think I have mastered the method of online learning.
	I feel competent in terms of high information technology literacy and will not encounter technical problems.
	I feel able to understand the structure and composition of the whole online learning platform.
	I think I have the ability to ensure the efficiency of online learning.
	I think I can actively participate in online learning and complete all online learning tasks.
Perceived autonomy	I think the content of the online learning courses is relevant to my interests.
	I can get timely feedback when I encounter difficulties or have queries.
	I think the content and resources of online learning are worth learning.
Perceived relatedness	The online learning course is closely related to my experience.
	Online learning courses have provided valuable help for my professional development.
	I experienced support from online learning that gives me a sense of friendliness, intimacy, and trust.
Well-being	Active
	Attentive
	Enthusiastic
	Excited
	Inspired
	Interested
	Determined
Satisfaction	I really enjoyed this form of online learning.
	I believe that participating in online learning can bring about improvements.
	I feel comfortable with the online learning method as a form of self-study.
	I would like to participate in various activities and tasks in online learning.
	I feel pleased and joyful to communicate with other students on the Internet.
	I feel happy and satisfied when I complete online learning successfully.
Behavioral intention	I would like to transfer my learning experience of online training to daily teaching.
	I can share resources and experience with other members throughout the online learning process.
	I want to know more about online learning in the future.
	I would like to use online learning every day after this online training program.
